# Airway Sensory Nerve Plasticity in Asthma and Chronic Cough

**DOI:** 10.3389/fphys.2021.720538

**Published:** 2021-09-07

**Authors:** Matthew G. Drake, Madeline Cook, Allison D. Fryer, David B. Jacoby, Gregory D. Scott

**Affiliations:** ^1^Division of Pulmonary and Critical Care Medicine, Department of Medicine, Oregon Health and Science University, Portland, OR, United States; ^2^Department of Pathology, Oregon Health and Science University, Portland, OR, United States

**Keywords:** asthma, cough, nerve, neurotrophin, eosinophil, P2X3, neurokinin

## Abstract

Airway sensory nerves detect a wide variety of chemical and mechanical stimuli, and relay signals to circuits within the brainstem that regulate breathing, cough, and bronchoconstriction. Recent advances in histological methods, single cell PCR analysis and transgenic mouse models have illuminated a remarkable degree of sensory nerve heterogeneity and have enabled an unprecedented ability to test the functional role of specific neuronal populations in healthy and diseased lungs. This review focuses on how neuronal plasticity contributes to development of two of the most common airway diseases, asthma and chronic cough, and discusses the therapeutic implications of emerging treatments that target airway sensory nerves.

## Introduction

Airway sensory nerves have essential roles in maintaining lung health and regulating airway physiology. Sensory nerves express a wide variety of receptors to detect inhaled and endogenous chemical and mechanical stimuli, and relay their input to second order neurons in the brainstem (McGovern and Mazzone, 2014). Brainstem neurons subsequently provide input to higher cortical pathways and to brainstem motor neurons that regulate reflexes such as breathing, cough, and bronchoconstriction. Sensory nerves also release neuropeptides, which have antimicrobial and immune modifying functions (McGovern et al., 2018). Recent advances have uncovered a host of homeostatic and adaptive sensory neuronal processes that maintain lung health or alternatively, contribute to the pathogenesis of disease. In this review, we discuss sensory nerve function in healthy lungs and explore how neuronal plasticity contributes to development of two of the most common airway diseases, asthma and chronic cough. Recent advances in therapies that target sensory nerves and new experimental techniques for studying nerve function will also be addressed.

## Sensory Nerve Structure and Function in Healthy Airways

**FIGURE 1:**
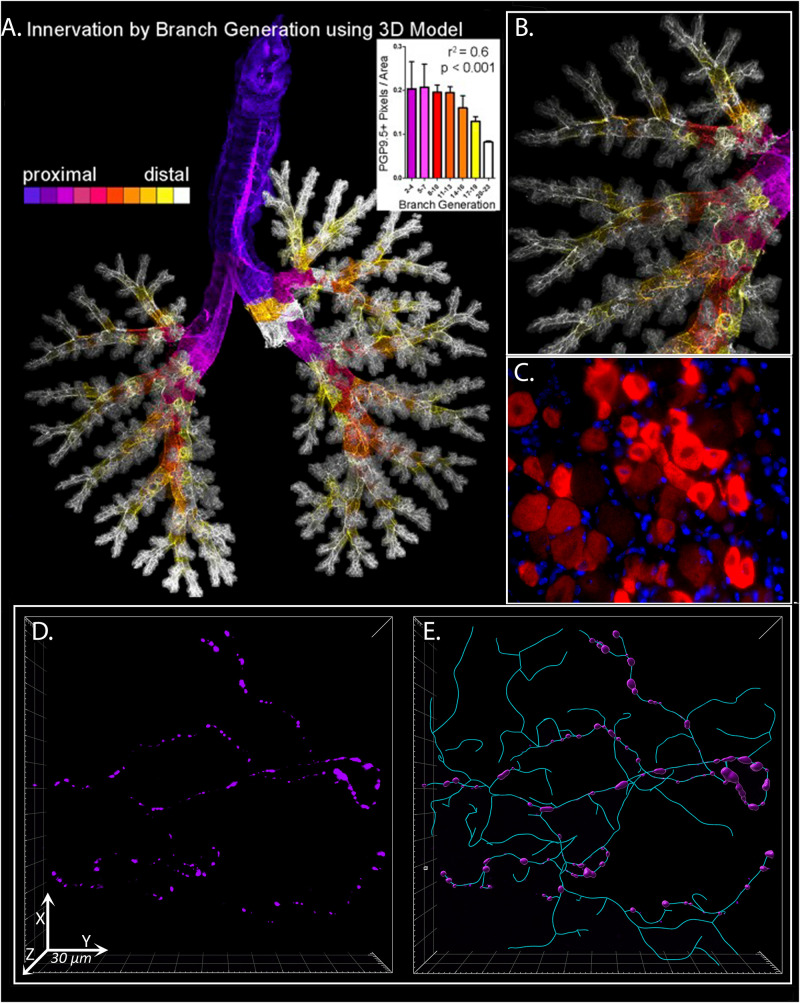
3D Analysis of Sensory Innervation of the Lung. Mouse and human airway nerves are labeled and imaged using immunofluorescence and confocal microscopy. **(A)** Flattened image of mouse lungs with nerves identified using antibodies against the pan-neuronal protein PGP9.5. Color-coding is based on airway generation from proximal (*purple*) to distal (*white*). Nerve density in each generation was calculated (*inset*). **(B)** Higher magnification image of airway shown in **(A)**. **(C)** Mouse vagal ganglion with purinergic P2X3 receptor expression in red. **(D)** Human airway neuronal substance P expression (purple) in bronchoscopic airway biopsy from a patient with chronic cough. **(E)** Computer modeling and morphometric analysis of total sensory innervation based on PGP9.5 (cyan) and distribution of substance P (purple) in image from **(D)**. **(A,B)** Scott et al. (2014) reprinted with permission of the American Thoracic Society. Copyright^©^ 2021 American Thoracic Society. All rights reserved. The American Journal of Respiratory Cell and Molecular Biology is an official journal of the American Thoracic Society. **(D,E)** From ref. Shapiro et al. (2021) adapted with permission of the American Thoracic Society. Copyright^©^ 2021 American Thoracic Society. All rights reserved. The American Journal of Respiratory and Critical Care Medicine is an official journal of the American Thoracic Society. Readers are encouraged to read the entire article for the correct context at https://www.atsjournals.org/doi/full/10.1164/rccm.201912-2347OC. The authors, editors, and The American Thoracic Society are not responsible for errors or omissions in adaptations.

Airway sensory nerves primarily originate in vagal ganglia at the base of the skull, with a small portion also supplied by dorsal root ganglia along the spine. Sensory axons terminate at all levels of airways, including in primary and secondary bronchi below and within the airway epithelium, smooth muscle, glands, and autonomic ganglia (Belvisi, 2002), and within the alveolar airspace distal to airways where they surround the capillary bed (Watanabe et al., 2006; Figure 1). Clusters of sensory nerve endings are particularly dense at branch points and in the dorsal aspects of airways (Scott et al., 2013).

Sensory neurons can be classified in a variety of ways based on their primary function, conduction speed, protein and peptide expression, or airway location. However, it is important to note that no single classification scheme fully encapsulates all these variables and that considerable overlap exists between the sensory properties of many nerves (Mazzone and Undem, 2016). Most commonly, sensory nerves are subdivided based on their ability to detect mechanical or chemical stimuli, termed mechanoreceptors or chemoreceptors, respectively. Mechanoreceptor are typically myelinated Aδ nerve fibers that are exquisitely sensitive to touch. Chemoreceptors (also termed nociceptors) are mostly unmyelinated fibers that are generally quiescent in healthy lungs, but express a wide array of receptors and ion channels capable of detecting inhaled or endogenous noxious compounds (Mazzone and Undem, 2016). Other airway afferent nerves include slowly adapting and rapidly adapting intrapulmonary mechanoreceptors that do not evoke cough directly, but can modify the function of cough pathways. Collectively, these sensory afferents contribute to the coordination of normal physiologic activity such as regulation of bronchoconstriction, breathing, and expulsion of foreign materials by stimulation of cough.

Sensory nerve afferents transmit information centrally to the solitary nucleus and paratrigeminal tract in the brainstem, which then relay signals to higher order neurons involved in cognition and sensation in the cerebral cortex, as well as other brainstem centers involved in unconscious processing of airway stimulation, including the respiratory central pattern generator and the nucleus ambiguus (Canning, 2009). Sensory input into the respiratory central pattern generator coordinates involuntary coughing, whereas sensory input to pre-ganglionic cholinergic parasympathetic nerves in the nucleus ambiguus triggers reflex bronchoconstriction (Costello et al., 1999). Reflex bronchoconstriction occurs when parasympathetic efferent axons contained within the vagal nerves stimulate release of acetylcholine from post-ganglionic parasympathetic nerves in the trachea and primary bronchi. Acetylcholine activates M_3_ muscarinic receptors on airway smooth muscle to trigger contraction and airway narrowing. Acetylcholine also activates prejunctional inhibitory M_2_ muscarinic receptors on airway parasympathetic nerves, which limit further acetylcholine release. This inhibitory feedback mechanism, coupled with inhibition of sensory reflexes by input from slow adapting mechanoreceptor neurons, helps regulate the degree of bronchoconstriction (Minette and Barnes, 1988; Fryer and Jacoby, 1992). Reflex bronchoconstriction occurs in response to a variety of stimuli, such as inhaled methacholine (Wagner and Jacoby, 1999), histamine (Boushey et al., 1980), cold air (Sheppard et al., 1982), allergens (Gold, 1973), and exercise (Simonsson et al., 1972), and is highly conserved in both humans and animal models.

In addition to the cholinergic nerve reflex, bronchodilatory efferent non-cholinergic parasympathetic and sympathetic neurons are also activated by sensory nerves, however, both their structure and function vary greatly between species. In humans, non-cholinergic parasympathetic nerves release peptides and neurotransmitters, including nitric oxide and vaso-intestinal peptide (VIP), which induce airway relaxation to counterbalance acetylcholine’s contractile effects (Richardson and Beland, 1976; Ward et al., 1995; Kesler et al., 2002). In contrast, airway sympathetic nerves are largely absent in humans, but present in many other animal species including dogs and guinea pigs (Richardson and Beland, 1976; Kummer et al., 1992). Sympathetic nerves induce airway relaxation via release of norepinephrine that activates beta receptors on smooth muscle. In humans, beta receptor activation occurs through effects of systemic norepinephrine rather than through airway sympathetic nerves specifically.

Sensory activation also leads to direct release of mediators locally within tissues via an axon reflex (Mazzone, 2005). Axon reflexes take place within a single sensory neuron, do not require central input, and involve direct release of small signaling peptides that are synthesized and secreted by sensory nerves. These neuropeptides comprise a large class of biologically active small proteins, including substance P, CGRP, and VIP among others, that modify nerve function, stimulate production of neuronal growth factors, and recruit inflammatory cells to airways (McGovern et al., 2018).

## Neurobiological Origins of Chronic Cough

Cough is a protective response that maintains healthy lungs by clearing pathogens, mucus, and environmental particles from the airways. Cough is also a common feature of over 100 different diseases, including most commonly, asthma, gastroesophageal reflux, rhinitis/upper airway cough syndrome, and during respiratory viral infections. In some cases, cough persists, lasting months or even years and no longer serves a physiologic role (Morice et al., 2020). Chronic cough is defined as any cough lasting more than 8 weeks. A common feature of patients with chronic cough is enhanced sensitivity to both tussive and non-tussive stimuli that develops via multiple mechanisms that contribute to neuronal sensitization, including increased sensitivity and expression of specific nociceptors on neurons, *de novo* expression of nociceptors by non-nociceptor neurons, increased epithelial nerve density, and increased release of endogenous cough-triggering molecules in airways. A variety of inflammatory mediators contribute to nerve sensitization, including leukotrienes, bradykinin, neurokinins, prostanoids, lipid mediators, and adenosine triphosphate, among others (Chung et al., 2013). Exposure to allergens, virus infection, cigarette smoke, ozone, and other insults, stimulates production and release of these mediators, sensitizing nerves and increasing cough frequency. Neuronal sensitization is one part of a spectrum of airway remodeling in chronic cough that includes airway epithelial damage, basement membrane thickening and in some cases, an influx of inflammatory cells such as mast cells or eosinophils (Morice et al., 2007). Just as inciting events and inflammatory mediators differ between diseases, so too does an individual’s response to various tussive agents. As a recent study demonstrated, patients with COPD and chronic idiopathic cough had similar cough sensitivity to inhaled capsaicin, but drastically differed in their sensitivity to inhaled prostaglandin E2 (Belvisi et al., 2016). Thus, different diseases exhibit unique cough neurophenotypes that manifest collectively as cough hypersensitivity. The following sections focus on specific features of neuronal sensitization.

### Transient Receptor Potential (TRP) Channels

TRP channels are calcium-permeable ion channels that trigger sensory nerve activation and are considered canonical cough receptors due to their ability to trigger cough in response to a diverse set of tussive stimuli. Foremost among these receptors are TRPV1, whose ligands include the active ingredient in chili powder, capsaicin, and citric acid; TRPA1, which responds to noxious compounds such as cinnamaldehyde, acrolein, and formalin; and TRPV4 which is activated by arachidonic acid and hypotonic solutions. Changes in both TRP expression and activity have been documented in chronic cough. For example, TRPV1 expression is increased in bronchial biopsies from patients with chronic cough (Groneberg et al., 2004; Mitchell et al., 2005), while *de novo* TRPV1 expression occurs in low-threshold mechanoreceptors after allergen exposure and virus infection in guinea pigs (Lieu et al., 2012; Zaccone et al., 2016) and after allergen exposure in rats (Zhang et al., 2008b, a). Similar changes in neuronal TRPV1 expression are induced by exposing isolated tracheas to the neurotrophin brain-derived neurotrophic factor (BDNF) (Lieu et al., 2012). Tobacco smokers, patients with asthma, and patients with chronic obstructive pulmonary disease (COPD) have increased responsiveness to TRPV1 agonists citric acid and capsaicin (Belvisi et al., 2016), supporting a role for TRP channels in cough hypersensitivity.

Several TRP channel antagonists have been developed for treatment of chronic cough and have proved quite effective at blocking cough responses to specific tussive agents (Khalid et al., 2014; Preti et al., 2015; Bonvini et al., 2016; Belvisi et al., 2017). However, these antagonists against TRPV1, TRPV4, TRPA1, and others have uniformly failed to improve spontaneous cough frequency or inhibit the urge to cough in chronic cough patients in clinical trials. These discordant outcomes suggest that changes in specific TRP receptors do not fully explain the neuronal mechanisms underlying cough hypersensitivity.

### Sensory Neuropeptides

Neurokinins, of which substance P is the most studied, are a large group of neuroactive peptides that have been associated with cough in several animal models, most notably in guinea pigs. Substance P, in particular, is constitutively expressed by small diameter nociceptive sensory nerves and is released via an axon reflex, causing both bronchoconstriction and potentiation of citric acid-induced cough (Moreaux et al., 2000; Otsuka et al., 2011; McGovern and Mazzone, 2014). Substance P expression can be induced *de novo* in larger diameter mechanoreceptors after allergen exposure and virus infection in guinea pigs (Carr et al., 2002; Myers et al., 2002), suggesting changes in neuropeptide content may contribute to nerve sensitization in chronic cough. Furthermore, blocking substance P receptors, termed neurokinin receptors, attenuates cough in guinea pigs (Girard et al., 1995).

Despite these promising results, substance P’s role in human cough remains unclear. Substance P was increased in peripheral blood of adults with chronic cough (Otsuka et al., 2011), but not in bronchoalveolar lavage fluid from children with cough (Chang et al., 2007). In bronchial biopsy specimens from adults with chronic cough, airway sensory nerve substance P expression was similar between chronic cough and healthy patients (O’Connell et al., 1995; Shapiro et al., 2021). Moreover, initial clinical trials of substance P receptor antagonists failed to improve cough frequency in patients with chronic cough (Fahy et al., 1995), adding to doubts that neuropeptides and substance P in particular are important for sensory nerve-mediated tussive responses. However, recent studies using newer neurokinin receptor antagonists that block receptors in both the central and peripheral nervous system reported a reduction in cough frequency in chronic cough patients (Smith J. et al., 2020), leading to renewed interest in this approach. Indeed, these findings suggest that substance P’s role in cough involves modulation of central neurotransmission between peripheral sensory nerves and second order brainstem neurons. This possibility, and the potential for targeting central effects of substance P using brain penetrant compounds, is the focus on ongoing studies.

### Neurotrophins

Neurotrophins encompass a large family of neuroactive molecules that influence sensory nerve development and acutely alter neuronal activity. Two of the most studied are nerve growth factor (NGF) and brain-derived neurotrophic factor (BDNF), which are released by inflammatory cells and by airway epithelium in the setting of allergen exposure, virus infection, and other insults (Prakash and Martin, 2014; Skaper, 2017). Both NGF and BDNF sensitize cough responses to TRPV1 agonists *in vivo* by increasing neuronal TRPV1 expression and by acutely enhancing TRPV1 channel activity (Ji et al., 2002; Zhang et al., 2005; Zhu and Oxford, 2007). NGF also enhances TRPA1 signaling (Clarke et al., 2017) and stimulates neuropeptide expression in sensory neurons (Hunter et al., 2000; Dinh et al., 2004). Moreover, mice that produce elevated levels of nerve growth factor from airway epithelium exhibit increased sensory nerve density (Hoyle et al., 1998). Increased nerve density is also present in mice exposed to allergen in early life due to production of another neurotrophin, neurotrophin-4 (Patel et al., 2016). Thus, a variety of neurotrophins may influence nerve phenotype, growth and function in the setting of airway inflammation. In adults with chronic cough, increased airway epithelial innervation is observed (Shapiro et al., 2021), although whether these changes occur during specific time periods of development and whether neurotrophins are involved has not been established.

### Purinergic Receptors

A subset of vagal sensory neurons express the purinergic receptor P2X3 (Wang et al., 2014). P2X3 binds ATP, an important endogenous alarmin released by both structural and inflammatory cells at times of cell stress, and triggers cough in humans and animals (Belvisi and Smith, 2017; Turner and Birring, 2019). Patients with chronic cough exhibit increased cough sensitivity to inhaled ATP (Fowles et al., 2017), suggesting receptor sensitization and/or changes in expression may occur. Increased extracellular ATP has also been reported in lung diseases that are associated with cough hypersensitivity, including asthma and COPD (Idzko et al., 2007; Lommatzsch et al., 2010), suggesting that increased P2X3 activation contributes to excessive cough in these disorders. Interestingly, a recent study also reported that TRPV4 stimulates neuronal ATP release (Bonvini et al., 2016). Thus, different cough receptors may work synergistically to increase cough frequency, although the clinical importance of this synergism is unclear given that a TRPV4 antagonist failed to improve cough frequency in a recent clinical trial (Ludbrook et al., 2019).

Clinical interest in P2X3 as a therapeutic target has recently risen due to trials showing the P2X3 antagonist gefapixant significantly reduced spontaneous cough frequency in patients with refractory or unexplained chronic cough (Abdulqawi et al., 2015; Morice et al., 2019; Smith J. A. et al., 2020). While this approach clearly offers the most promising treatment opportunity for chronic cough to date, it is notable that not all patients responded to gefapixant, suggesting there remains a need for improved neurophenotyping to better identify subsets of patients who are likely to respond to treatment. Indeed, gefapixant did not prevent cough provoked by inhaled capsaicin, indicating that TRPV1 and P2X3 induced coughing represent distinct mechanisms that need additional clinical characterization.

## Neuro-Immune Interactions in Asthma

Asthma is an inflammatory airway disease characterized by increased bronchoconstriction and airway hyperreactivity, where inhaled irritants provoke excessive airway contractions (Busse, 2010). Both increased bronchoconstriction and airway hyperreactivity are manifestations of sensory nerve dysfunction. Thus, like chronic cough, asthma can be viewed as neuropathic disease. Airway inflammatory cells in asthma, of which eosinophils are the most common in approximately two-thirds of patients, increase sensory nerve activation by altering nerve phenotype and increasing nerve density, and by provoking release of mediators that stimulate nerves directly, such as prostaglandins, histamine, thromboxanes, and tachykinins (Drake et al., 2018a). Eosinophils preferentially migrate to nerves due to neuronal expression of eosinophil chemoattractant factors, such as eotaxin-1, which binds to CCR3 to stimulate eosinophil chemotaxis (Griffiths-Johnson et al., 1993). In guinea pigs, neuronal eotaxin-1 expression is increased after allergen challenge and eosinophil migration to nerves and nerve-mediated airway hyperreactivity is blocked by CCR3 antagonists (Fryer et al., 2006) and by corticosteroids (Nie et al., 2007). Eosinophils also cause dysfunction of parasympathetic nerves after exposure to the environmental pollutant ozone (Yost et al., 2005) and after respiratory virus infections (Nie et al., 2009), both of which are common triggers for asthma exacerbations. In guinea pigs, blocking TNF-α after ozone exposure or virus infections prevents eosinophil recruitment to nerves and nerve-mediated airway hyperreactivity (Nie et al., 2011; Wicher et al., 2017). Thus, physical interactions between eosinophils and airway nerves are critical for development of nerve dysfunction in multiple models of asthma.

### Epithelial Sensory Nerve Density

Airway remodeling is a hallmark of longstanding asthma. Changes include airway smooth muscle hypertrophy, mucus gland hypersecretion, thickening of the basement membrane and epithelial metaplasia. Sensory nerves are no exception. Recently, airway epithelial sensory nerves were imaged using confocal microscopy in bronchial biopsies from patients with asthma (Drake et al., 2018b). In asthmatic samples, sensory nerves density was significantly increased compared to patients without airway disease. These changes were particularly apparent in patients with higher eosinophil counts and were associated with worse lung function and increased sensitivity to environmental irritants, suggesting nerve density contributes to classic features of the asthma phenotype. Similar increases in nerve density were present in transgenic mice with airway eosinophilia due to over-expression of interleukin-5 (IL-5), which promotes eosinophil recruitment and survival, and increased nerve density potentiated reflex bronchoconstriction triggered by inhaled serotonin (Drake et al., 2018b). Mice that were eosinophil-deficient did not develop airway hyperinnervation nor airway hyperreactivity, highlighting the important role for eosinophils in sensory neuronal remodeling.

### Sensory Neuropeptides

Sensory neuropeptides are increased in bronchoalveolar lavage fluid of patients with asthma (Nieber et al., 1992), and increased substance P-expressing sensory fibers are present in airway epithelium in bronchial biopsies from eosinophilic asthmatics (Drake et al., 2018b). In animal models, airway inflammation, and eosinophils in particular, stimulate neuropeptide expression in airways. For example, neuronal substance P expression is increased after allergen (Fischer et al., 1996) and ozone exposure (Wu et al., 2001) and in the setting of respiratory virus infections in guinea pigs (Carr et al., 2002). While these changes are clearly a consequence of inflammation, evidence suggests neuropeptides promote inflammation as well. Eosinophils migrate along a neuropeptide concentration gradient *in vitro* (Dunzendorfer et al., 1998), which may contribute to disproportionate numbers of eosinophils found clustered around airway nerves in asthmatics who suffered from fatal bronchoconstriction (Costello et al., 1997). Neuropeptides stimulate eosinophil entry into tissue by upregulating the adhesion molecules ICAM-1, VCAM-1, and E-selectin on vascular endothelium (Smith et al., 1993; Nakagawa et al., 1995; Quinlan et al., 1999), resulting in enhanced eosinophil migration into airway parenchyma, and ICAM-1 and VCAM-1 on nerves (Sawatzky et al., 2002), enabling eosinophil binding directly to nerves via their cognate receptors, VLA-4 and CD11b, respectively. In turn, blocking VLA-4 prevents eosinophil-mediated nerve dysfunction after allergen inhalation in guinea pigs (Fryer et al., 1997). Ligation of adhesion molecules on eosinophils has multiple effects on eosinophil function including release of leukotrienes and highly charged granule proteins eosinophil cationic protein and eosinophil peroxidase, which activate nerves directly and lower their activation threshold for other stimuli (Gu et al., 2008, 2009). Thus, neuropeptides and eosinophils interact to form a positive feedback loop, where neuropeptides and eosinophils cooperatively increase each other’s expression and activity, resulting in increased nerve sensitivity.

### TRP Channels

TRP channel agonists, such as capsaicin, induce bronchoconstriction in the absence of airway inflammation (Lalloo et al., 1995). In severe asthma, TRPV1 expression is increased (McGarvey et al., 2014) and TRPV1 responses are potentiated (Belvisi et al., 2016). In guinea pigs, exposure to ovalbumin antigen acutely sensitizes sensory nerve responsiveness to capsaicin while also decreasing the force necessary to activate mechanoreceptor neurons (Lieu et al., 2012). Inhalation of inflammatory mediators such as bradykinin and prostaglandin E2 similarly sensitized responses to capsaicin in healthy volunteers (Choudry et al., 1989), while eosinophil-derived cationic granule proteins increased isolated sensory nerve responses to capsaicin *in vitro* (Gu et al., 2008, 2009).

Like neuropeptides, TRP channels may also be involved in airway inflammation. Genetic silencing of TRPV1-expressing neurons in mice attenuated antigen-induced airway hyperreactivity, while stimulation of TRPV1-expressing neurons potentiated antigen-induced airway hyperreactivity *in vivo* (Trankner et al., 2014). Similar results were observed with TRPA1, where loss of TRPA1 function due to genetic knockdown or pharmacologic inhibition attenuated neuropeptide release and prevented airway hyperreactivity in mice (Caceres et al., 2009). Therefore, TRP channels may serve a key role in the interactions between neurons and airway inflammation that leads to hyperreactive asthmatic airway responses.

### Neurotrophins

Neurotrophins, including NGF, BDNF, and neurotrophin 3, are increased in asthma and these factors may have important roles in neuroplasticity (Bonini et al., 1996; Virchow et al., 1998; Olgart Hoglund et al., 2002; Watanabe et al., 2015). A variety of structural cells release neurotrophins, including airway neurons, smooth muscle and epithelium (Kistemaker and Prakash, 2019), which is a rich source of neurotrophins and is also the site of sensory nerve remodeling in both cough and asthma (Drake et al., 2018b; Shapiro et al., 2021). Inflammatory cells, such as mast cells and eosinophils also release neurotrophins, and in the case of allergic asthma, eosinophil-specific NGF release is increased (Raap et al., 2008b).

Neurotrophins activate tropomyosin-related tyrosine kinase (Trk) receptors on sensory nerves to induce structural, phenotypic and functional changes in nerve signaling. Specifically, NGF over-expression in transgenic mice increases airway nerve density (Hoyle et al., 1993), increases substance P expression in sensory ganglia (Dinh et al., 2004) and stimulates phenotypic switching in non-nociceptive fibers toward a nociceptor phenotype (Hunter et al., 2000). NGF also prolongs eosinophil survival (Raap et al., 2008a) and causes airway hyperreactivity *in vivo* (Verbout et al., 2007). In turn, blocking NGF, either with pharmacologic antagonists or by genetic silencing, attenuates airway hyperreactivity (Verbout et al., 2007; Verhein et al., 2011; Chen et al., 2014). Similarly, BDNF induces TRPV1 expression *de novo* in sensory neurons and increases their responsiveness to TRPV1 agonists (Lieu et al., 2012), while genetic modification of the BDNF receptor, TrkB, attenuates airway hyperreactivity and reduces nerve density (Aven et al., 2014). The effects of blocking these neurotrophins and receptors await further testing in humans.

## Sensory Neuronal Plasticity During Development

There is increasing evidence that critical developmental time-periods exist where sensory nerves are particularly vulnerable to the effects of various exposures. For example, exposure in very early life to ozone or allergen increased airway epithelial sensory innervation in rhesus monkeys (Kajekar et al., 2007), while ozone exposure in the early post-natal period in rats similarly produced increased epithelial nerve density (Hunter et al., 2010). Infections in early life by viruses such as respiratory syncytial virus (RSV) are also implicated in alternations in nerve development. RSV increases airway hyperreactivity in children and has been shown experimentally to alter neuronal control of bronchoconstriction in ferrets (Larsen and Colasurdo, 1999). RSV increases NGF, which may initiate the developmental effects on airway nerves that have long-lasting implications for airway function.

The prenatal period, during which exposures can influence airway nerve development in the fetus, poses another important time period for asthma risk. Evidence of these effects includes the observations that airway hyperreactivity can be detected at birth (Haland et al., 2006) and that children born to parents with asthma are at increased risk of developing asthma (Paaso et al., 2014). Furthermore, maternal asthma confers greater risk than paternal asthma (Kelly et al., 1995), supporting the concept that asthma risk is related in part to *in utero* exposures and not solely due to shared genetics or environmental exposures after birth.

Recently, Lebold et al. (2019) demonstrated that wild-type offspring of interleukin-5 (IL-5) transgenic mice, who are exposed to high levels of the eosinophil maturation cytokine IL-5 *in utero*, exhibit increased airway sensory innervation and reflex bronchoconstriction in later life. These effects are mediated by IL5’s induction of fetal eosinophilia. Importantly, these mice also have an exaggerated inflammatory response to allergen exposure in adulthood that results in airway hyperreactivity so severe that inhaled serotonin provokes lethal bronchoconstriction. Similar exposure to IL-5 and other asthma-related cytokines may occur in human fetuses born to asthmatic mothers as well. Thus, prenatal programming due to exposures *in utero* may create a unique trajectory that influences neuronal development and inflammatory responses to allergen in later life.

## Novel Methods for Studying Airway Nerves

Recent studies have illuminated a remarkable degree of heterogeneity in airway sensory nerve expression, structure and function. For example, Kupari et al. (2019) demonstrated using single cell RNA sequencing that 18 transcriptionally distinct sensory nerve subtypes exist within the vagal ganglia. Individual receptors and neuropeptides were often expressed by multiple subtypes despite each subset having fundamentally different functions in the airway. These results highlight the limitations of categorization efforts that grouped nerves based solely on protein expression and underscore the need for linking comprehensive expression profiling to nerve function. Indeed, even relatively rare nerve subtypes may provide prominent input for control of airway function, as is the case with MrgprC11 expressing nerves, which despite comprising only 2–6% of sensory neurons, contribute to control of cholinergic bronchoconstriction (Han et al., 2018). In total, these studies reinforce the need for advanced experimental techniques that allow targeted, specific analysis of infrequent or rare nerve subtypes and their receptors.

To this end, several studies recently demonstrated the utility of optogenetics for testing effects of specific pulmonary nerve subtype activation. Optogenetics is a technique where the light-activated ion channel, channelrhodopsin, is expressed in specific neurons using cre-lox recombination, enabling activation of neuronal subtypes using pulsed light. This technique has been applied to test airway contractions induced by cholinergic airway neurons (Pincus et al., 2020), to differentiate vagal neurons involved in control of breathing (Chang et al., 2015; Nonomura et al., 2017), to show that light activate TRPV1-S1PR3 expressing neurons are involved in airway hyperresponsiveness after antigen challenge (Trankner et al., 2014), and to identify a rare subset of P2RY1-expressing laryngeal sensory nerves that coordinate upper airway responses to protect against aspiration (Prescott et al., 2020). Optogenetic techniques have potential to greatly advance our understanding of neuronal subtype function *in vivo* in future studies.

Until recently, studies of nerve morphology have been limited by conventional 2 dimensional histological assessments of tissue section that has led to an underappreciation of the rich neural supply lungs receive. Sensory nerves form complex, three-dimensional branching structures in airway epithelium that can span 100’s of histologic tissue sections yet comprising only a small percentage of the total airway surface area (West et al., 2015). Individual nerves frequently innervate multiple, spatially distinct receptive fields (Yu and Zhang, 2004). Moreover, nerve density is not uniform across the airways, but rather, is increased at airway branch points (i.e., carina, bifurcation of major bronchi) and in the posterior large airways (Scott et al., 2013). The sporadic nature of neuronal features, further accentuated by the non-uniform expression of neuropeptides and other proteins by nerves, make 2 dimensional tissue section analyses prone to large sampling error. To overcome these limitations, our group has recently used tissue optical clearing and confocal microscopy to perform detailed quantitative analyses of nerve structure and expression in whole mount airway samples (Figure 1; Scott et al., 2014). Indeed, our studies have demonstrated both an remarkable degree of structural complexity in airway epithelial nerves and identified a significant increase in nerve density in airway of patients with eosinophilic asthma (Drake et al., 2018b) as well as patients with idiopathic chronic cough (Shapiro et al., 2021).

Combining targeted functional assessments with high resolution 3D microscopy offers exciting new opportunities for studying the structure and function of specific nerve subtypes. Interactions between immune cells and airway nerves, as is seen in eosinophilic asthma, can also be quantified. Thus, these techniques may provide new mechanistic insights into the role of airway nerves in disease that can be translated into new therapeutic targets and may lead to improved disease phenotyping.

## Conclusion

Airway sensory afferents have a central role in regulation of airway physiology and maintenance of lung health. Recent advancements in 3D confocal imaging, single cell PCR, and cre-lox techniques for testing specific nerve subtype activation have potential to provide new mechanistic insights into nerve function in health and disease. These studies may be harnessed to identify or refine disease phenotypes and to identify new therapeutic opportunities for patients. These advancements, coupled with the recent success of neuronally-targeted therapies such as P2X3 and NK1 receptor antagonists, suggest sensory nerves will have a prominent position in therapeutic development in the years ahead.

## Author Contributions

MD wrote the manuscript. MC, AF, DJ, and GS provided critical input and revisions to the final manuscript. All authors read and approved the final manuscript.

## Conflict of Interest

MD reports consulting fees for Astra Zeneca and GSK. The remaining authors declare that the research was conducted in the absence of any commercial or financial relationships that could be construed as a potential conflict of interest.

## Publisher’s Note

All claims expressed in this article are solely those of the authors and do not necessarily represent those of their affiliated organizations, or those of the publisher, the editors and the reviewers. Any product that may be evaluated in this article, or claim that may be made by its manufacturer, is not guaranteed or endorsed by the publisher.
